# Supplementary figures and images for: Genomic Surveillance of Clinical Pseudomonas aeruginosa Isolates Reveals an Additive Effect of Carbapenemase Production on Carbapenem Resistance
Source: Microbiol Spectr. 2022 May 31;10(3):e00766-22. doi: 10.1128/spectrum.00766-22 (PMC9241860; doi:10.1128/spectrum.00766-22)

**A**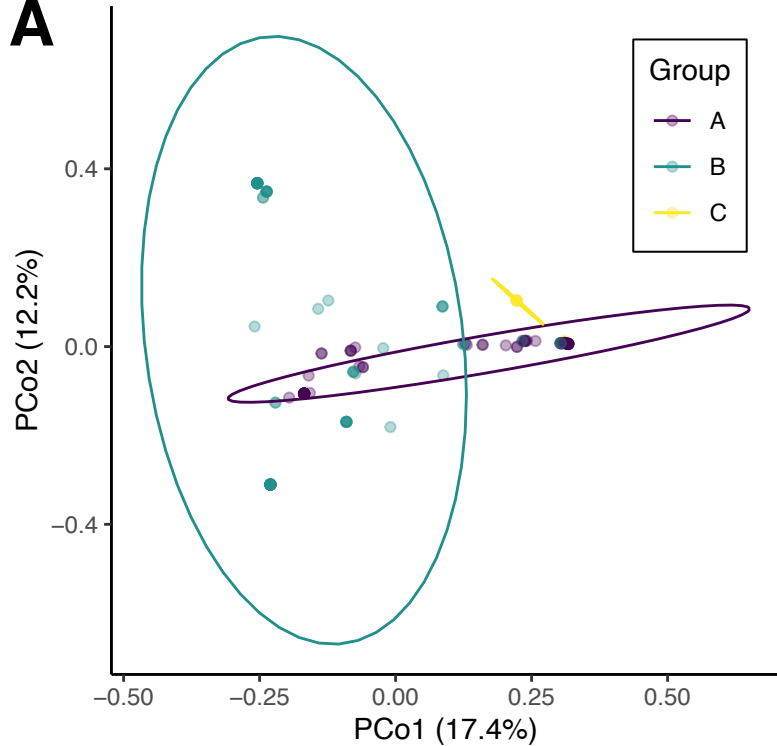**B**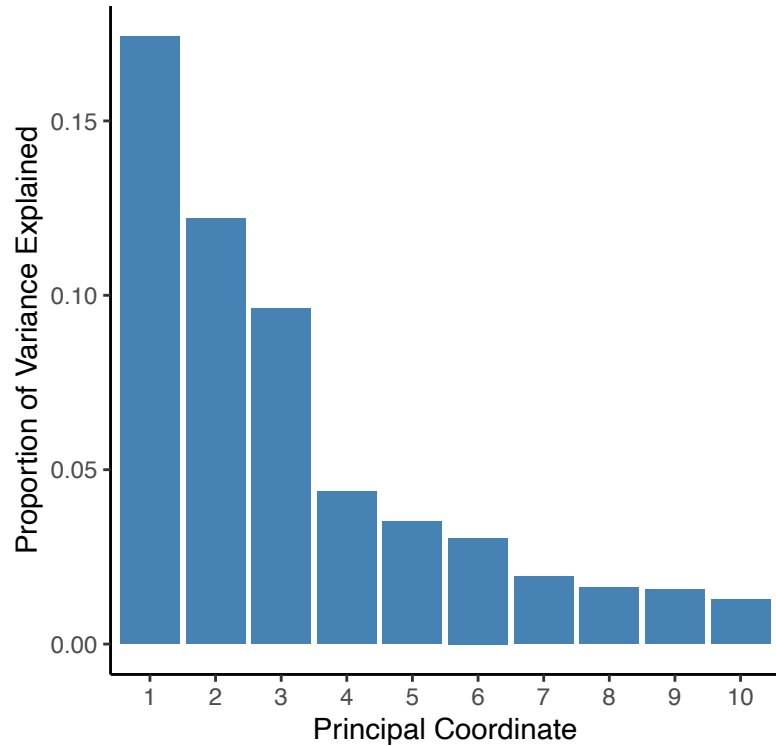

Supplement: Supplemental file 2 — Supplemental material. Download spectrum.00766-22-s0002.pdf, PDF file, 0.2 MB. [file spectrum.00766-22-s0002.pdf]

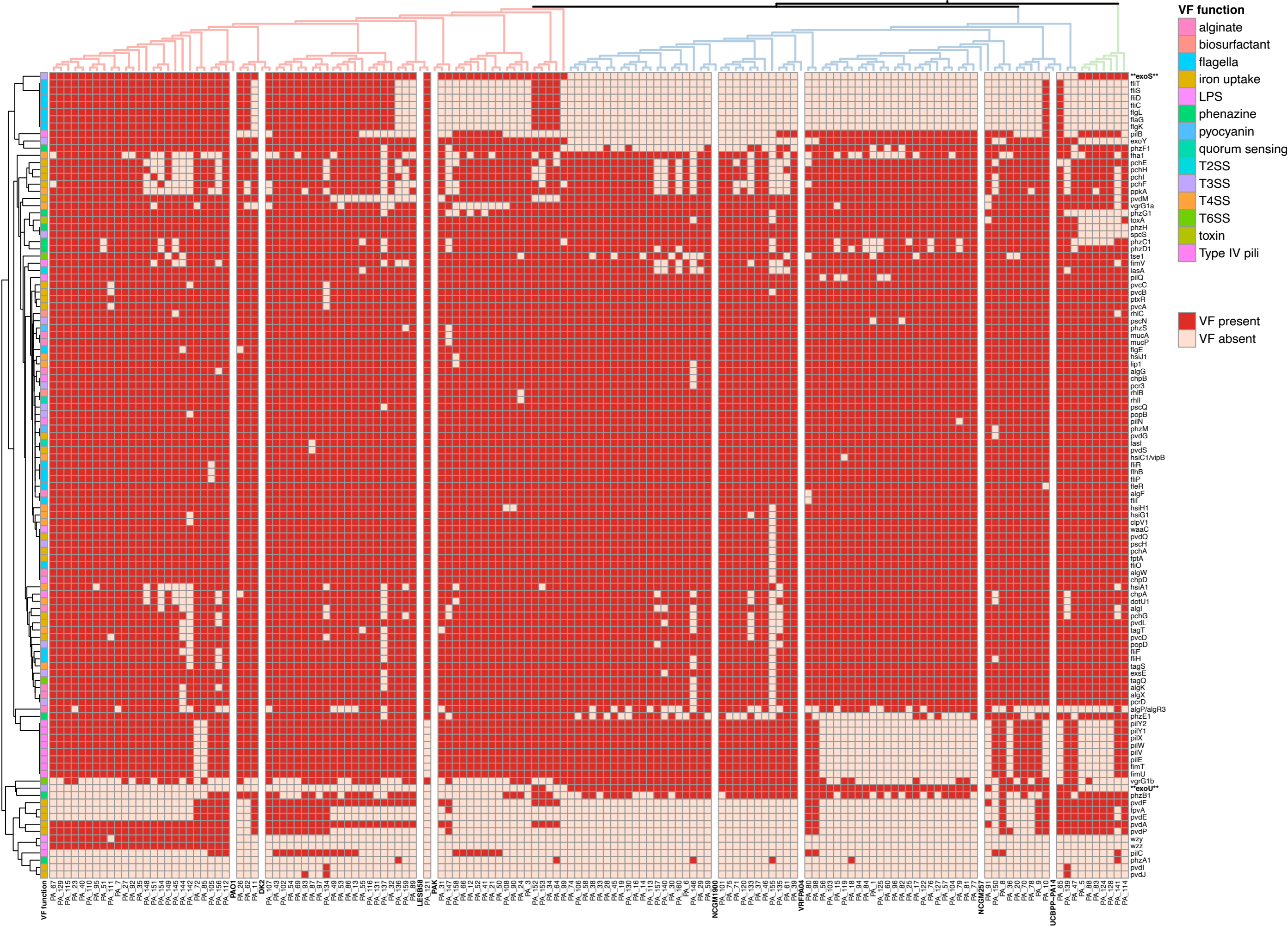

Supplement: Supplemental file 3 — Supplemental material. Download spectrum.00766-22-s0003.pdf, PDF file, 0.2 MB. [file spectrum.00766-22-s0003.pdf]

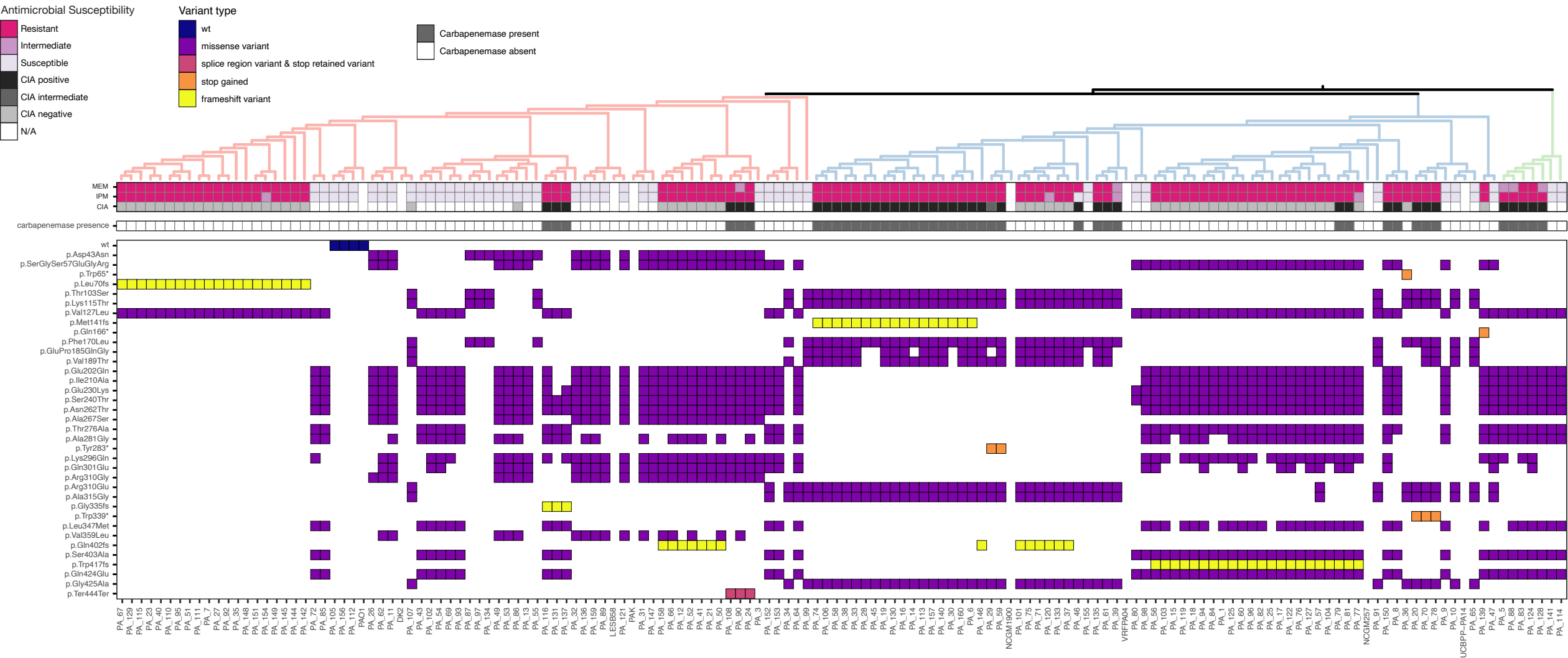

Supplement: Supplemental file 4 — Supplemental material. Download spectrum.00766-22-s0004.pdf, PDF file, 0.2 MB. [file spectrum.00766-22-s0004.pdf]

**A**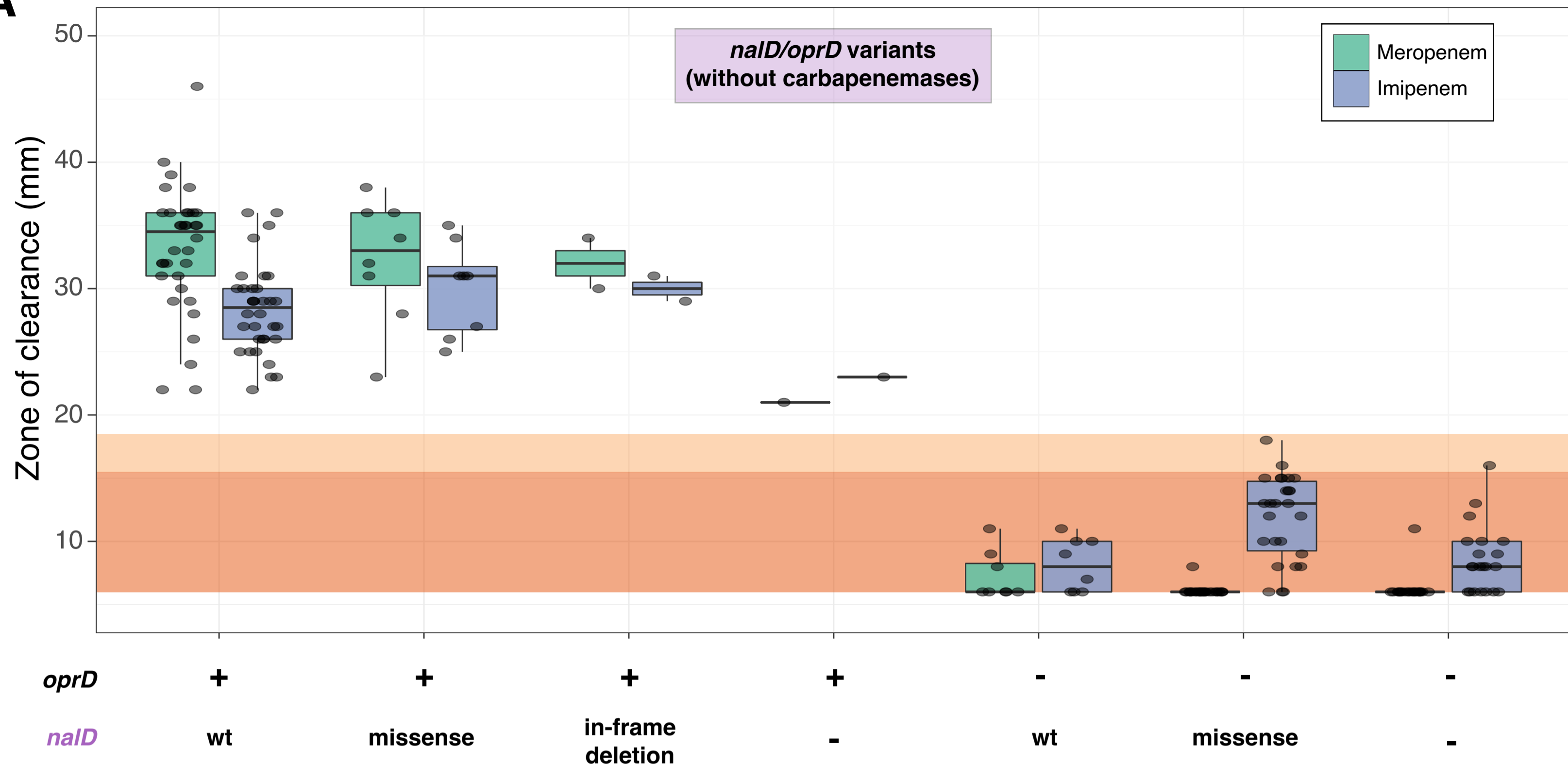**B**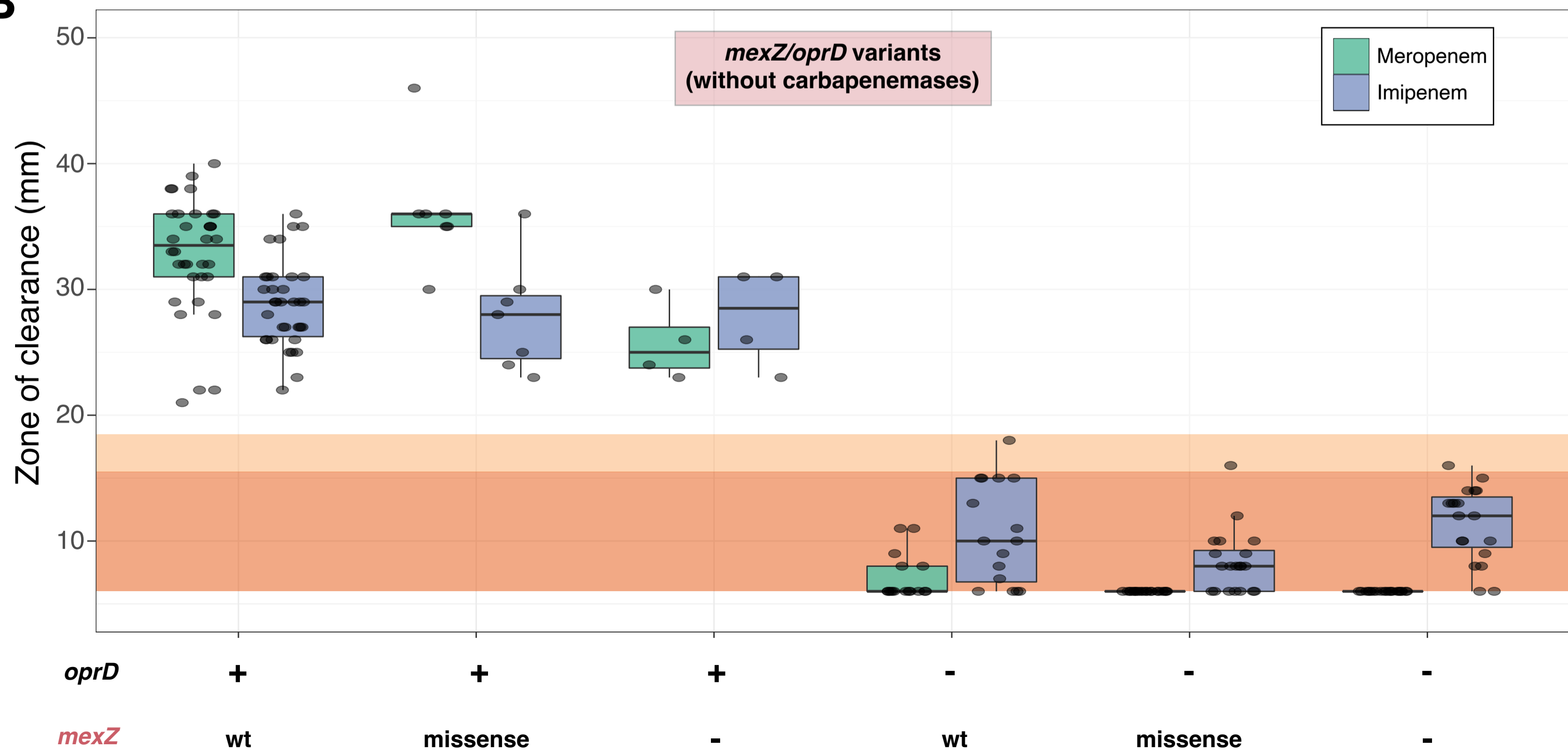

Supplement: Supplemental file 5 — Supplemental material. Download spectrum.00766-22-s0005.pdf, PDF file, 0.7 MB. [file spectrum.00766-22-s0005.pdf]

PA\_1,  
contig 69

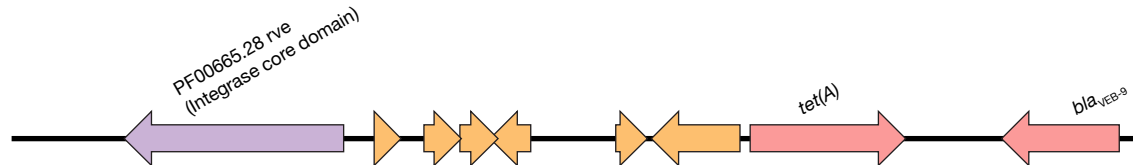

1 Kbp

PA\_116,  
contig 50

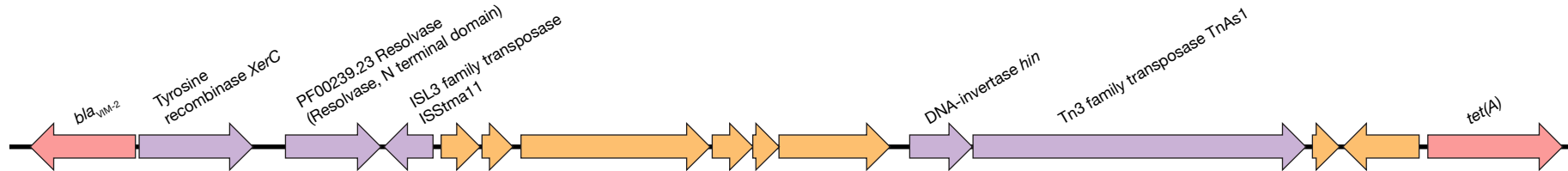

PA\_146,  
contig 234

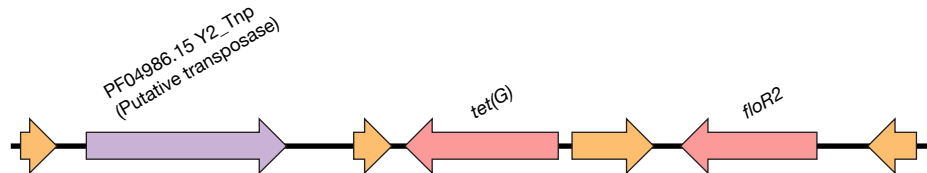

annotation:  ARG  MGE  other

Supplement: Supplemental file 6 — Supplemental material. Download spectrum.00766-22-s0006.pdf, PDF file, 0.1 MB. [file spectrum.00766-22-s0006.pdf]
