# Supplementary material for: Genomic Surveillance of Clinical Pseudomonas aeruginosa Isolates Reveals an Additive Effect of Carbapenemase Production on Carbapenem Resistance
Source: Microbiol Spectr. 2022 May 31;10(3):e00766-22. doi: 10.1128/spectrum.00766-22 (PMC9241860; doi:10.1128/spectrum.00766-22)
Supplement: Supplemental file 1 — Supplemental material. Download spectrum.00766-22-s0001.pdf, PDF file, 0.5 MB. [file spectrum.00766-22-s0001.pdf]

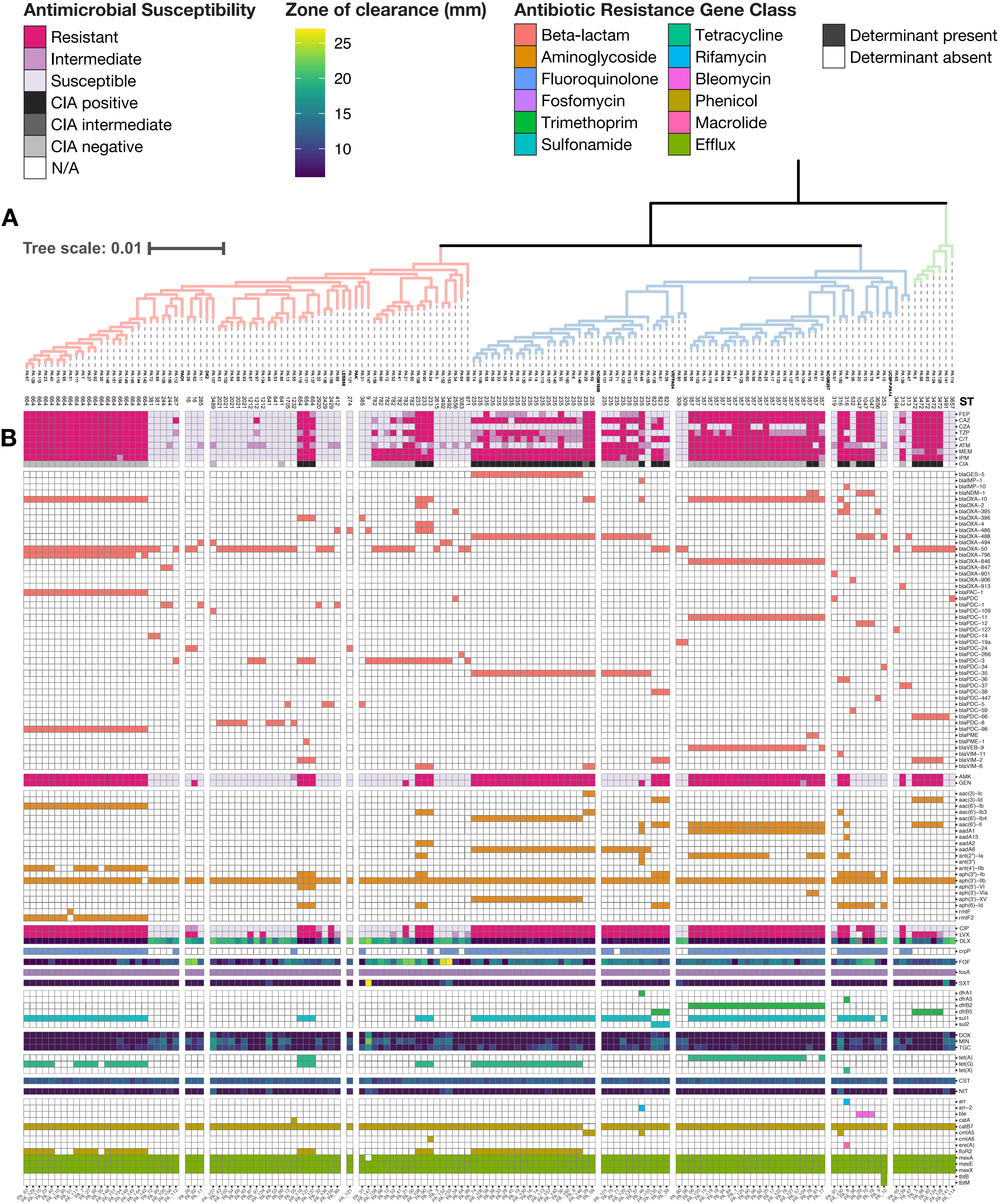

Antimicrobial Susceptibility
Resistant
Intermediate
Susceptible
CIA positive
CIA intermediate
CIA negative
N/A
Zone of clearance (mm)
25
20
15
10
Antibiotic Resistance Gene Class
Beta-lactam
Aminoglycoside
Fluoroquinolone
Fosfomycin
Trimethoprim
Sulfonamide
Tetracycline
Rifamycin
Bleomycin
Phenicol
Macrolide
Efflux
Determinant present
Determinant absent
A
Tree scale: 0.01
B
ST
FEP
CAZ
CZA
TZP
C/T
ATM
MEM
IPM
CIA
AMK
GEN
CIP
LVX
DLX
crpP
FOF
fosA
SXT
dfrA1
dfrA5
dfrB2
dfrB5
sul1
sul2
DOX
MIN
TGC
tet(A)
tet(G)
tet(X)
CST
NIT
arr
arr-2
ble
catA
catB7
cmlA5
cmlA6
ere(A)
floR2
mexA
mexE
mexX
tbtB
tbtM
